# Artificial intelligence in the diagnosis of necrotising enterocolitis from abdominal radiographs: a systematic review and meta-analysis

**DOI:** 10.3389/fped.2026.1852254

**Published:** 2026-06-18

**Authors:** Vivek Mahapatra, Aishwarya Chandra, Tanveer Rehman, Srijeeta Mitra, Prajina Pradhan, Asha Kiran, Sunil Kumar Agarwalla, Sanghamitra Pati

**Affiliations:** 1 Department of Paediatrics, Sardar Vallabh Bhai Patel Post Graduate Institute of Paediatrics, SCB Medical College and Hospital, Cuttack, Odisha, India; 2 ICMR Regional Medical Research Centre, Bhubaneswar, Odisha, India; 3 Model Rural Health Research Unit, Namkum, Ranchi, Jharkhand, India; 4 Department of Radiology, Sum Ultimate Medicare, Bhubaneswar, Odisha, India; 5 Department of Community Medicine, Rajendra Institute of Medical Sciences, Ranchi, Jharkhand, India; 6 Indian Council of Medical Research Headquarters, New Delhi, India

**Keywords:** abdominal radiography, artificial intelligence, machine learning, necrotizing enterocolitis, neonate

## Abstract

**Context:**

Necrotising enterocolitis (NEC) is a leading cause of morbidity and mortality in premature and very low birth weight neonates. Early diagnosis is challenging as clinical and laboratory features are non-specific. Artificial intelligence (AI) offers a potential means to improve diagnostic consistency and timeliness.

**Objective:**

To systematically review and meta-analyse the diagnostic accuracy of AI-based models for identifying NEC from abdominal radiographs.

**Evidence acquisition:**

A systematic search of MEDLINE, Embase, CINAHL, IEEE Xplore, and the Cochrane Library was conducted for studies published between January 1, 2015, and July 20, 2025. Studies evaluating AI, machine learning, or deep learning models applied to abdominal radiographs for NEC diagnosis or stratification, and reporting diagnostic performance metrics, were included. Study selection, data extraction, and risk-of-bias assessment (modified PROBAST) were performed independently by two reviewers. Diagnostic accuracy was pooled using hierarchical summary receiver operating characteristic models.

**Results:**

Ten retrospective studies met inclusion criteria; six were eligible for meta-analysis. Most employed convolutional neural networks, with limited external validation. The pooled sensitivity for AI-based diagnosis of NEC was 0.78 (95% CI 0.67–0.85) and pooled specificity was 0.84 (95% CI 0.73–0.91), with substantial heterogeneity. Positive and negative likelihood ratios were 4.84 and 0.27, respectively, indicating moderate diagnostic value. Explainability analyses commonly highlighted clinically relevant bowel features.

**Conclusion:**

AI-based interpretation of abdominal radiographs demonstrates moderate accuracy for NEC diagnosis and may serve as a decision-support adjunct rather than a standalone test. Clinicians should view these tools as complementary aids within existing diagnostic frameworks, pending prospective validation of AI models and standardised implementation into existing workflows.

**Systematic Review Registration:**

https://www.crd.york.ac.uk/PROSPERO/view/CRD420251090229.

## Introduction

Necrotising enterocolitis (NEC), a severe gastrointestinal emergencies, predominantly affects premature and very low birth weight (VLBW) infants (1). Globally, NEC occurs in 5%–10% of VLBW neonates, with mortality rates ranging from 23% to over 50% in surgically managed cases (2). Despite advances in neonatal intensive care, NEC remains responsible for nearly 10% of neonatal deaths (3), and contributes substantially to long-term morbidity, including intestinal failure, neurodevelopmental impairment, and prolonged hospitalisation (2). Early identification and accurate risk stratification are critical, as timely medical escalation or surgical intervention can be lifesaving and may mitigate irreversible intestinal injury (4). The modified Bell's staging criteria, continue to serve as the cornerstone for NEC diagnosis and staging in clinical practice and research (5). These criteria integrate systemic signs, gastrointestinal manifestations, laboratory abnormalities, and radiological findings. However, early-stage NEC (Bell stage I) is characterised by non-specific clinical and laboratory features that frequently overlap with neonatal sepsis, feeding intolerance, and spontaneous intestinal perforation. This diagnostic ambiguity often delays definitive management and contributes to inter-clinician variability (6, 7).

Abdominal radiography (AR/AXR) remains the primary imaging modality for evaluating suspected NEC and is core component of the modified Bell's criteria. Classic radiographic findings, such as pneumatosis intestinalis, portal venous gas, fixed dilated bowel loops, and pneumoperitoneum, are highly specific for NEC (7, 8). Identifying these signs is challenging. A study estimated that about 28% of radiology trainees could correctly identify pneumatosis on radiographs (9). Pneumatosis intestinalis is pathognomic for NEC, but other diagnoses may also exhibit this sign (10, 11). Sensitivity is limited, at around 44%, as early radiographic changes may be subtle (12). Interpretation of AXRs is inherently subjective and prone to inter- and intra-observer variability (7, 9, 13). Despite these limitations, abdominal radiography remains indispensable due to its widespread availability, rapid acquisition, low cost, and feasibility for serial monitoring. AXRs allow longitudinal assessment of disease progression, bowel gas patterns, and complications such as perforation (8). Abdominal ultrasound and standardised radiographic scoring systems, have been proposed to enhance diagnostic accuracy and detect earlier signs of NEC, such as bowel wall thickening and perfusion abnormalities (13). However, ultrasound is highly operator-dependent, lacks universal standardisation, and are not readily available in all neonatal units, limiting its scalability.

Artificial intelligence (AI), particularly machine learning (ML) and deep learning (DL), has demonstrated substantial promise in medical image interpretation (14, 15). AI-based systems have achieved diagnostic performance comparable to expert clinicians in detecting diabetic retinopathy from fundus photographs, classifying pulmonary tuberculosis and pneumonia from chest radiographs, identifying intracranial haemorrhage on computed tomography, and detecting malignancies in histopathology and radiology images (15–19). Meta-analyses across multiple imaging have reported pooled sensitivities and specificities frequently exceeding 80%, highlighting the capacity of AI algorithms to recognise complex spatial patterns (14). In neonatology, AI applications have increasingly focused on prediction models incorporating clinical and physiological data to estimate NEC risk. However, heterogeneity in input variables, model architectures, outcome definitions, and validation strategies has limited their generalisability and clinical adoption (20). Few studies have focused specifically on AI-based detection of NEC from abdominal radiographs, despite AXR being universally performed, and central to diagnostic frameworks. To address this evidence gap, we aimed to systematically synthesise and evaluate the diagnostic accuracy of AI-based models for identifying NEC from abdominal radiographs in neonates.

## Methods

### Study design and registration

We conducted a systematic review and meta-analysis of original studies evaluating the diagnostic accuracy of AI and ML methods for detecting NEC from AXRs. The review followed the PRISMA-DTA guidelines and was prospectively registered with PROSPERO (CRD420251090229) on 20th July 2025 to ensure methodological transparency.

### Search strategy

A comprehensive literature search was conducted across five electronic databases: MEDLINE (via PubMed), CINAHL (via EBSCO), Embase (via Ovid), IEEE Xplore, and the Cochrane Library. The initial search was conducted on July 24, 2025, with an update on August 1, 2025. The search strategy was designed to identify studies that developed and/or validated AI-based algorithms for diagnosing NEC using abdominal radiographs, and, where applicable, compared algorithmic performance with healthcare professionals. The search combined Medical Subject Headings (MeSH), Emtree terms, and keywords, including “artificial intelligence”, “machine learning”, “necrotising enterocolitis”, “diagnosis”, “abdominal radiograph”, and “abdominal x-ray”. Advanced Boolean operators (“AND” and “OR”) were utilised. The search was refined based on preliminary screening and in consultation with a systematic review expert (TR). The search included peer-reviewed articles published in English between January 1, 2015, and July 20, 2025. Reference lists of included studies were also screened manually. A detailed search strategy is provided in the Supplementary File.

### Search eligibility

Studies were included if they met the following criterias:

- Employed AI, ML, or DL methods to AXR for NEC diagnosis
- Classified radiographs into at least one clinically relevant outcome, including NEC vs. no pathology, or surgical NEC vs. medical NEC vs. no pathology
- Reported diagnostic performance metrics [e.g., sensitivity, specificity, area under the receiver operating characteristic curve (AUC), accuracy].
- Used any reference standard for NEC diagnosis, including clinical diagnosis, expert consensus, or surgical findings.

Eligible study designs included observational studies (cross-sectional, case–control, cohort studies) and interventional studies. We excluded review articles, editorials, letters, guidelines, expert opinions, conference abstracts without full peer-reviewed manuscripts; studies limited to image segmentation, feature extraction, or radiomics without diagnostic classification.

### Study selection

Search results were imported into Covidence software for screening and data extraction. After removing duplicates, studies underwent a two-stage screening process. In the Titles and abstracts were independently screened by two reviewers (VM and AC). The Cohen's kappa coefficient for inter-rater agreement was 0.7, indicating substantial agreement. Discrepancies were resolved through discussion with the senior reviewer (TR). Shortlisted studies underwent full-text screening by same researchers to confirm eligibility. The Cohen's kappa coefficient for full-text screening was 0.8.

### Data extraction

Data extraction was performed independently by two reviewers (VM and AC) using a pre-tested, standardised data extraction form in Microsoft Excel. Extracted data included: author, year of publication, country, study setting, study design, AI model architecture, sample size, population characteristics; number of radiographs allocated to training, tuning, validation, and test datasets; NEC definition and staging criteria; reference standard used for outcome ascertainment; comparator (healthcare professional interpretation, if applicable); and reported diagnostic performance metrics (sensitivity, specificity, AUC, accuracy, *F*1 score, precision, recall, positive predictive value, negative predictive value, C-index). Data were cross-verified by a third reviewer (TR), and disagreements were resolved by consensus. Corresponding authors were contacted up to three times over four weeks, till 27 August 2025 to obtain missing information. Studies were retained if key outcomes were available despite unresolved missing data.

### Statistical analysis

We constructed contingency tables at reported diagnostic thresholds and calculated sensitivity and specificity. Meta-analyses were conducted for studies providing internally validated diagnostic accuracy estimates. Given anticipated heterogeneity, pooled estimates were generated using hierarchical models. Unified hierarchical summary receiver operating characteristic (HSROC) model was applied to estimate pooled sensitivity and specificity and construct summary ROC curves. HSROC plots included 95% confidence regions and 95% prediction regions to illustrate between-study heterogeneity. Likelihood ratios (LRs) were calculated with 95% confidence intervals (CI). Statistical heterogeneity was assessed using the chi-square (*Q*) test and quantified using *I*^2^ statistic, with values of 25%, 50%, and 75% indicating low, moderate, and high heterogeneity, respectively. A two-sided *p* value of <0.05 was considered statistically significant. Analyses were performed using Stata version 16 (StataCorp, College Station, TX) with the “midas” and “metandi” modules.

### Quality assessment

A modified version of Prediction Model Risk of Bias Assessment Tool (PROBAST) was used to assess the risk of bias (RoB) of included studies. It comprises of 20 signalling questions across four domains: participants, predictors, outcomes, and analysis. Two reviewers (VM and AC) assessed each study, and disagreements were resolved through discussion. Responses were recorded as “yes”, “no” or “unclear” and each domain was rated as having low, high, or unclear RoB. The overall RoB for a study was considered low, only when all domains had low risk.

### Publication bias

To minimise publication bias, we searched multiple databases spanning clinical medicine and engineering disciplines, screened reference lists of included studies, and reviewed relevant preprint servers. Publication bias was performed using regression-based methods examining asymmetry in diagnostic log odds ratios (OR).

## Results

### Study selection

**Figure 1:**
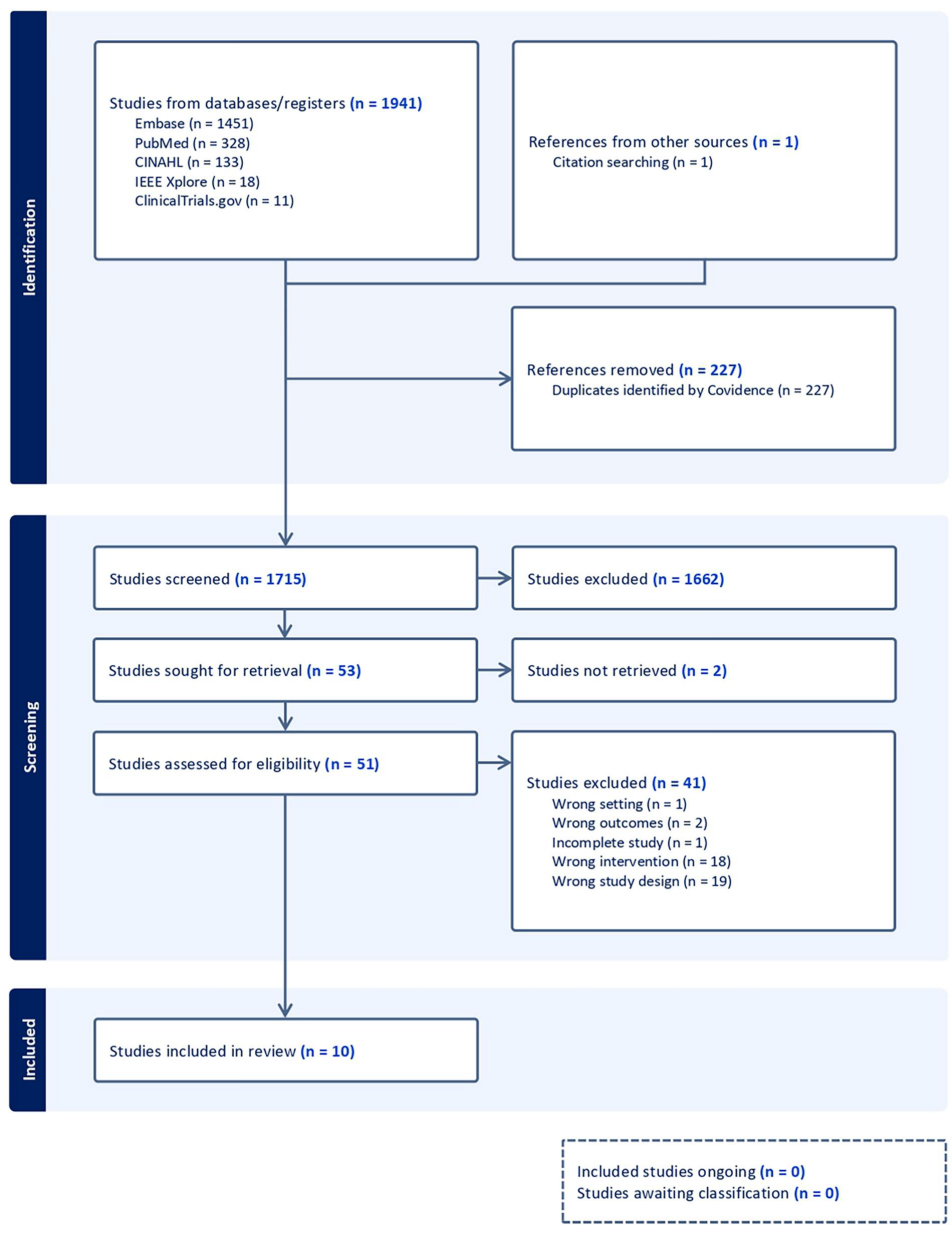
PRISMA flow diagram illustrating the study selection process of included studies.

Database search identified 1,942 records. After duplicates removal, 1,715 studies underwent title and abstract screening, of which 1,662 were excluded as clearly irrelevant. Fifty-three articles were retrieved for full-text assessment; full texts could not be obtained for two studies. Of the remaining 51 articles, 41 were excluded. Ten studies met the eligibility criteria and were included in the qualitative synthesis, of which six provided sufficient data for meta-analysis. The study selection process is summarised in the PRISMA flow diagram (Figure 1).

### Study characteristics

All included studies employed retrospective designs and evaluated AI-based methods for diagnosing NEC or predicting surgical requirement using abdominal radiographs or radiology reports. Studies were published between 2018 and 2025 and originated from three geographic regions: China (*n* = 5), the United States (*n* = 3), and the United Kingdom (*n* = 2). No prospective or randomised diagnostic accuracy studies were identified. Across the ten studies, over 14,000 abdominal radiographs, radiology reports, or associated records were analysed. Most studies analysed the radiograph taken at the initial diagnosis of NEC (21–23). In evaluating surgical NEC, the last radiograph before taking up for surgery was analysed (23, 24). Some studies used established NEC databases with radiographs containing representative findings (25–27). Image-based DL architectures predominated, most commonly convolutional neural networks using ResNet backbones (ResNet-34, ResNet-50), SENet-154, and transformer-based models. Three studies incorporated radiomics approaches, and two employed natural language processing (NLP) or large language models (LLMs) to extract diagnostic labels from unstructured radiology reports. Internal validation was performed in seven studies using held-out test sets or cross-validation. One study reported temporal validation, while none conducted external multicentre validation. Comparator performance from healthcare professionals was reported in one study. Baseline characteristics and study outcomes are illustrated in Table 1.

**Table 1:** Baseline characteristics and performance comparison of the included studies of AI-based models for diagnosing NEC from abdominal radiographs.

Author	Country	Model type	No. of patients	Gestational age in weeks (mean)	Gender: male *n* (%)	NEC diagnosed *n* (%)	Definition of NEC (reference standard)	*F*1 score	Accuracy	Precision	Recall	AUC	Sensitivity	Specificity	PPV	NPV
Diagnosis of NEC																
Cui et al. (21)	China	Res-Net34	408	33	198 (49%)	204 (50%)	Bell	0.8493	0.9199	0.8606			0.9145	0.8668		
Lu et al. (22)	China	Radionomics	484	33.4	281 (58%)	262 (54%)	Bell		0.64			0.74 (0.64–0.83)	0.57	0.71	0.67	
Nowak et al. (26)	UK	Augmented ResNet 50	382	35	225 (59%)	239 (62%)	Bell	0.693	0.693	0.7	0.693					
Weller et al. (25)	USA	ResNet 50	494	34.5			Pneumatosis +		0.878			0.918 (0.837–0.978)	0.762	0.964		
Gao et al. (28)	China	SunNet-154	827		494 (53%)	342 (41%)	Bell		0.8434 (0.7983–0.8885)	0.9371 (0.9069–0.9673		0.8757 (0.8347–0.9166)	0.8542 (0.8104–0.8980)	0.807 (0.7580–0.8560)		
Yung KW et al. (27)	UK	AIDNEC	334			194 (58%)	Bell	0.747	0.797	0.742	0.758					
Zhang et al. (30)	USA	Gemma-7b-it					Pneumatosis/ PVG +	0.9 (0.015)								
Crowley et al. (29)	USA	RRA					Pneumatosis/ PVG +					0.94				
Prediction of surgical NEC																
Li et al. (23)	China	XGBoost	171	33	65 (38%)	171 (100%)	Bell		0.92			0.959	0.85	0.95	0.85	0.95
Gao et al. (28)	China	SenNET-154	379	34.2	231 (61%)	379 (100%)	Bell		0.8455 (0.7816–0.9090)	0.8861 (0.8300–0.9422)		0.8198 (0.7519–0.8877)	0.875 (0.8166–0.9334)	0.7907 (0.7188–0.8626)		
Wu et al. (24)	China	ResNet-18	263	32.9		263 (100%)	Bell	0.824	0.85	0.933	0.737	0.876 (0.766–0.986)	0.737	0.952	0.933	0.8
Yung KW et al. (27)	UK	AIDNEC	334			194 (58%)	Bell	0.678	0.694	0.688	0.686					

UK, United Kingdom; USA, United States of America; NEC, necrotising enterocolitis; AUC, area under curve; PPV, positive predictive value; NPV, negative predictive value.

### Diagnosis of NEC

Eight studies evaluated AI-based methods for diagnosing NEC. Five studies developed DL image classification models, two used NLP or LLM-based approaches applied to radiology reports, and one used a radiomics-only diagnostic model. Among image-based studies, 2,929 neonates were included, with mean sample size of 488 per study. Mean gestational age, reported in four studies, ranged from 33.0 to 34.5 weeks. Training datasets comprised 246–1,743 images, while test sets ranged from 49 to 254 images.

Several studies emphasised interpretability. Cui et al. demonstrated that ResNet-34 model focused on clinically meaningful radiographic features, including fixed bowel loops and portal venous gas, using Grad-CAM visualisation (21). Lu et al. developed a radiomics model using 18 handcrafted features extracted from abdominal regions including liver to capture portal venous gas (22). Nowak et al. reported that targeted preprocessing and augmentation pipelines improved model performance for subtle NEC findings in a three-class classification task (surgical NEC vs. medical NEC vs. no pathology) (26). Weller et al. trained a ResNet-50 classifier to detect pneumatosis with performance comparable to senior surgical residents; however, the use of publicly available, curated images enriched for advanced disease raised concerns regarding spectrum bias (25). Gao et al. identified SENet-154 as the optimal architecture following pretraining on over 4,500 abdominal radiographs and demonstrated its utility both as standalone model and feature extractor for multimodal integration (28). Yung et al. developed the AIDNEC model using fine-grained visual classification techniques, enabling automated localisation of discriminative regions without explicit annotations (27).

### NLP and LLM-based diagnosis

Two studies evaluated automated NEC diagnosis using radiology report text. Crowley et al. analysed 6,239 radiology reports from four neonatal intensive care units using a rule-based NLP tool to identify NEC-related findings, highlighting reduced generalisability across institutions due to linguistic variability (29). Zhang et al. fine-tuned a privacy-preserving LLM (Gemma-7B) to identify NEC directly from unstructured reports (30).

### Prediction of surgical NEC

Four studies evaluated AI-based stratification of NEC into surgical vs. medical disease. Three used DL image classifiers, and one employed radiomics-based machine learning models (23, 24, 27, 28). Across these studies, 1,147 neonates were included, with training datasets ranging from 119 to 792 images and test sets from 40 to 254 images. Radiomics-based models showed moderate discriminatory ability for predicting surgical intervention, particularly using ensemble models such as XGBoost. DL studies reported that heatmap-based explainability methods consistently highlighted bowel regions associated with disease severity. Two studies reported both diagnosis and surgical prediction performance within the same modelling framework (27, 28).

### Risk of bias and quality assessment

Using the modified PROBAST tool, six studies were judged to have low overall RoB, while four had unclear to high risk, primarily due to small sample sizes, lack of external validation, and potential spectrum bias. The analysis domain was the most common source of concern, reflecting overfitting risks and limited reporting of calibration. A summary of the RoB assessment is presented in Supplementary Figure S1.

### Meta-analysis of diagnostic accuracy

**Figure 2:**
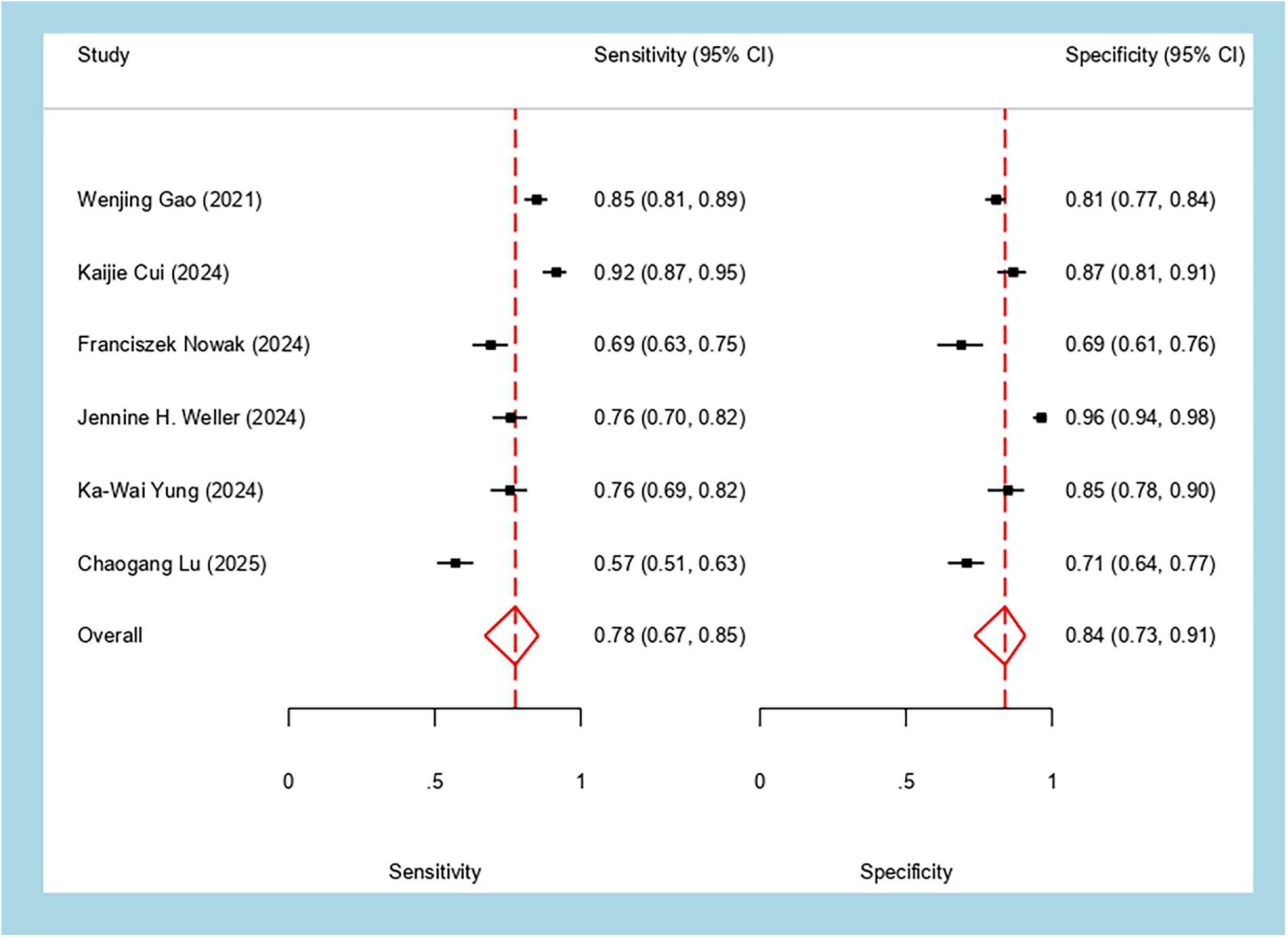
Forest plot of pooled sensitivity and specificity of AI-based models for diagnosing NEC from abdominal radiographs.

Six studies provided sufficient data for quantitative synthesis. Two studies with incomplete contingency tables required imputation using standard methods (26, 27). For studies reporting multiple models, the best-performing model was included. As external validation was inconsistent, test set performance was used for pooling. The pooled sensitivity of AI-based methods for diagnosing NEC from abdominal radiographs was 0.78 (95% CI 0.67–0.85), with substantial heterogeneity (*Q* = 106.8, *p* < 0.001; *I*^2^ = 93.5%) (Figure 2). The pooled specificity was 0.84 (95% CI 0.73–0.91), also with high heterogeneity (*Q* = 81.76, *p* < 0.001; *I*^2^ = 91.4%). The HSROC curve demonstrated wide prediction regions, indicating considerable between-study variability (Supplementary Figure S2). The pooled positive LR was 4.84 (95% CI 2.68–8.72), and the pooled negative LR was 0.27 (95% CI 0.17–0.42). The diagnostic OR was 18.14 (95% CI 6.92–47.59). These findings suggest, AI-based interpretation of abdominal radiographs provides moderate diagnostic value but is insufficient as a standalone rule-in or rule-out test for NEC (Supplementary File).

### Publication bias

No evidence of publication bias was detected on regression-based assessment of diagnostic log OR (bias coefficient −25.18, 95% CI −258.15 to 207.79; *p* = 0.78) (Supplementary File).

## Discussion

This review synthesised available evidence on the diagnostic performance of AI-based models for detecting NEC from abdominal radiographs. The pooled diagnostic accuracy estimates provide the first quantitative benchmark for radiograph-based AI models in NEC.

Relative to well-described diseases like cancer and sepsis, few studies have explored the utility of AI and ML models in NEC (20). These methods have been used for biomarker discovery, prediction of NEC or its outcomes, distinguishing NEC from spontaneous intestinal perforation, evaluating treatment options and current NEC definitions (20).

Earlier linear and multiple regression models were used. With gradual increase in sophistication, they progressed to supervised ML methods (31, 32). The early studies were based on Electronic Medical Record (EMR) data, and incorporated radiological findings documented by doctors. Optimising these models focused on identifying the minimum number of variables, while preserving diagnostic accuracy.

However, these models often struggled when data was missing. EMRs may store data differently, but affects the generalizability. With advances in DL models, multiple studies have explored the prediction of NEC from abdominal x-rays (21, 25–28).

Some studies train multi-modal models by integrating an image processing predictor with a clinic-laboratory predictor (21, 28). We posit that it may be useful to focus on a radiological model to minimise the limitations of missing data. This may be the first attempt to synthesise the available data on this topic.

Weller et al., compared a radiological model for pneumatosis diagnosis with senior surgical residents and reported comparable AUC scores (25). Gao et al. designed a multimodal model that performed on par with senior-level clinicians in diagnosing NEC, and even exceeded them in predicting surgical NEC (28). Integration of the clinical predictive model improved the diagnostic accuracy metrics of the radiological model by 5%–8%.

Our study shows that radiological models demonstrate good pooled sensitivity and specificity for diagnosing NEC. However, NEC diagnosis rarely depends on a single radiograph, and serial imaging is often required as radiographic findings evolve over time. Model performance may therefore improve in longitudinal clinical use. This is important, considering that a radiological model addresses the limitation of missing data, which is prominent in multi-modal or clinico-laboratory model.

Several studies incorporated explainability techniques highlighting clinically plausible regions such as bowel loops and portal venous structures. Such transparency is likely essential for clinician trust, regulatory approval, and medico-legal accountability in decision-support systems.

While radiological models are unlikely to replace clinical decision-making, their simplicity, robustness to missing data, and alignment with existing diagnostic workflows may make them deployable clinical decision-support tools.

Our study has certain limitations. First, the number of eligible studies was small, limiting statistical power and precision of pooled estimates. Two studies required derivation of missing performance data using standard methods, introducing potential uncertainty. Potential confounders exist in both radiograph acquisition and clinical diagnosis. As most studies were retrospective, variability in the timing and quality of bedside radiographs may have influenced model performance. Additionally, NEC is a clinically heterogeneous condition, and the initial suspicion prompting radiography is often clinician-dependent, introducing variability into case identification and labelling.

Substantial heterogeneity observed is unsurprising given the differences in NEC definitions, radiograph acquisition protocols, model parameters, and validation strategies. Future studies should prioritise harmonised definitions and standardised reporting of calibration and decision curve metrics. Additionally, the lack of external validation across studies restricts generalisability.

## Conclusion

AI and ML methods showed a moderate diagnostic accuracy in diagnosing NEC from abdominal radiographs, with a pooled sensitivity of 0.78 and specificity of 0.84. They offer a pragmatic advantage by mitigating the impact of missing data, and show promise as deployable assistant tools to aid clinicians.

## Data Availability

The original contributions presented in the study are included in the article/Supplementary Material, further inquiries can be directed to the corresponding author.

## References

[1] Alsaied A Islam N Thalib L. Global incidence of necrotizing enterocolitis: a systematic review and meta- analysis. BMC Pediatr. (2020) 20(1):344. 10.1186/s12887-020-02231-5 32660457 PMC7359006

[2] Jones IH Hall NJ. Contemporary outcomes for infants with necrotizing enterocolitis-a systematic review. J Pediatr. (2020) 220:86–92.e3. 10.1016/j.jpeds.2019.11.011 31982088

[3] Jacob J Kamitsuka M Clark RH Kelleher AS Spitzer AR. Etiologies of NICU deaths. Pediatrics. (2015) 135:e59–65. 10.1542/peds.2014-2967 25489010

[4] Bethell GS Knight M Hall NJ. Surgical necrotizing enterocolitis: association between surgical indication, timing, and outcomes. J Pediatr Surg. (2021) 56:1785–90. 10.1016/j.jpedsurg.2021.04.028 34090670

[5] Walsh MC Kliegman RM. Necrotizing enterocolitis: treatment based on staging criteria. Pediatr Clin North Am. (1986) 33:179–201. 10.1016/S0031-3955(16)34975-6 3081865 PMC7131118

[6] Di Napoli A Di Lallo D Perucci CA Schifano P Orzalesi M Franco F. Inter-observer reliability of radiological signs of necrotising enterocolitis in a population of high-risk newborns. Paediatr Perinat Epidemiol. (2004) 18:80–7. 10.1111/j.1365-3016.2003.00517.x 14738550

[7] Coursey CA Hollingsworth CL Gaca AM Maxfield C DeLong D Bisset G. Radiologists’ agreement when using a 10-point scale to report abdominal radiographic findings of necrotizing enterocolitis in neonates and infants. Am J Roentgenol. (2008) 191:190–7. 10.2214/AJR.07.3558 18562745

[8] Coursey CA Hollingsworth CL Wriston C Beam C Rice H Bisset G. Radiographic predictors of disease severity in neonates and infants with necrotizing enterocolitis. Am J Roentgenol. (2009) 193:1408–13. 10.2214/AJR.08.2306 19843760

[9] Sharma PG Rajderkar DA Sistrom CL Slater RM Mancuso AA. Bubbles in the belly: how well do radiology trainees recognize pneumatosis in pediatric patients on plain film? Br J Radiol. (2022) 95:20211101. 10.1259/bjr.20211101 35073159 PMC9153714

[10] Hoehn T Stöver B Bührer C. Colonic pneumatosis intestinalis in preterm infants: different to necrotising enterocolitis with a more benign course? Eur J Pediatr. (2001) 160:369–71. 10.1007/s004310100757 11421417

[11] Coşkun Y Özen MA Kayaş K Arıkan Ç Gürsoy T. Pneumatosis intestinalis: does it always indicate necrotizing enterocolitis? Turk J Pediatr. (2024) 66:768–74. 10.24953/turkjpediatr.2024.5308 39807732

[12] Tam AL Camberos A Applebaum H. Surgical decision making in necrotizing enterocolitis and focal intestinal perforation: predictive value of radiologic findings. J Pediatr Surg. (2002) 37:1688–91. 10.1053/jpsu.2002.36696 12483631

[13] Esposito F Mamone R Di Serafino M Mercogliano C Vitale V Vallone G. Diagnostic imaging features of necrotizing enterocolitis: a narrative review. Quant Imaging Med Surg. (2017) 7:336–44. 10.21037/qims.2017.03.01 28812000 PMC5537125

[14] Aggarwal R Sounderajah V Martin G Ting DSW Karthikesalingam A King D. Diagnostic accuracy of deep learning in medical imaging: a systematic review and meta-analysis. NPJ Digit Med. (2021) 4:65. 10.1038/s41746-021-00438-z 33828217 PMC8027892

[15] Liu X Faes L Kale AU Wagner SK Fu DJ Bruynseels A. A comparison of deep learning performance against health-care professionals in detecting diseases from medical imaging: a systematic review and meta-analysis. Lancet Digit Heal. (2019) 1:e271–97. 10.1016/S2589-7500(19)30123-2 33323251

[16] Chilamkurthy S Ghosh R Tanamala S Biviji M Campeau NG Venugopal VK. Deep learning algorithms for detection of critical findings in head CT scans: a retrospective study. Lancet. (2018) 392:2388–96. 10.1016/S0140-6736(18)31645-3 30318264

[17] Gulshan V Peng L Coram M Stumpe MC Wu D Narayanaswamy A. Development and validation of a deep learning algorithm for detection of diabetic retinopathy in retinal fundus photographs. J Am Med Assoc. (2016) 316:2402–10. 10.1001/jama.2016.17216 27898976

[18] Lakhani P Sundaram B. Deep learning at chest radiography: automated classification of pulmonary Tuberculosis by using convolutional neural networks. Radiology. (2017) 284:574–82. 10.1148/radiol.2017162326 28436741

[19] Rajpurkar P Irvin J Zhu K Yang B Mehta H Duan T. CheXNet: radiologist-level pneumonia detection on chest x-rays with deep learning (2017). Available online at: https://arxiv.org/pdf/1711.05225 (Accessed January 24, 2026).

[20] McElroy SJ Lueschow SR. State of the art review on machine learning and artificial intelligence in the study of neonatal necrotizing enterocolitis. Front Pediatr. (2023) 11:1182597. 10.3389/fped.2023.1182597 37303753 PMC10250644

[21] Cui K Changrong S Maomin Y Hui Z Xiuxiang L. Development of an artificial intelligence-based multimodal model for assisting in the diagnosis of necrotizing enterocolitis in newborns: a retrospective study. Front Pediatr. (2024) 12:1388320. 10.3389/fped.2024.1388320 38827221 PMC11140039

[22] Lu C Yang M Zhu Y Xia Y Luo S Yang G. Evaluation of radiomics as an assistant tool for radiologists in the diagnosis of necrotizing enterocolitis. Transl Pediatr. (2025) 14:559–70. 10.21037/tp-2024-496 40386358 PMC12079682

[23] Li Y Wu K Yang H Wang J Chen Q Ding X. Surgical prediction of neonatal necrotizing enterocolitis based on radiomics and clinical information. Abdom Radiol. (2024) 49:1020–30. 10.1007/s00261-023-04157-9 38285178

[24] Wu Z Zhuo R Liu X Wu B Wang J. Enhancing surgical decision-making in NEC with ResNet18: a deep learning approach to predict the need for surgery through x-ray image analysis. Front Pediatr. (2024) 12:1405780. 10.3389/fped.2024.1405780 38895195 PMC11183801

[25] Weller JH Scheese D Tragesser C Yi PH Alaish SM Hackam DJ. Artificial intelligence vs. Doctors: diagnosing necrotizing enterocolitis on abdominal radiographs. J Pediatr Surg. (2024) 59:161592. 10.1016/j.jpedsurg.2024.06.001 38955625 PMC11401766

[26] Nowak F Yung KW Sivaraj J De Coppi P Stoyanov D Loukogeorgakis S. An investigation into augmentation and preprocessing for optimising x-ray classification in limited datasets: a case study on necrotising enterocolitis. Int J Comput Assist Radiol Surg. (2024) 19:1223–31. 10.1007/s11548-024-03107-0 38652416 PMC11178627

[27] Yung KW Sivaraj J Coppi D Stoyanov P Loukogeorgakis D Mazomenos S. Diagnosing necrotizing enterocolitis via fine-grained visual classification. IEEE Trans Biomed Eng. (2024) 71:3160–9. 10.1109/TBME.2024.3409642 39453790

[28] Gao W Pei Y Liang H Lv J Chen J Zhong W. Multimodal AI system for the rapid diagnosis and surgical prediction of necrotizing enterocolitis. IEEE Access. (2021) 9:51050–64. 10.1109/ACCESS.2021.3069191

[29] Crowley PA Brociner E Uppili H Kohane I. Ultra-high accuracy in artificial intelligence-based classification of radiology reports from a necrotizing enterocolitis database: results from a cross-institutional investigation. Espghan and Naspghan (2018) p. 67.

[30] Zhang Y Kohne JG Webster K Vartanian R Wittrup E Najarian K. AXpert: human expert facilitated privacy-preserving large language models for abdominal x-ray report labeling. JAMIA Open. (2025) 8. 10.1093/jamiaopen/ooaf008 PMC11809431 39931456

[31] Uauy RD Fanaroff AA Korones SB Phillips EA Phillips JB Wright LL. Necrotizing enterocolitis in very low birth weight infants: biodemographic and clinical correlates. J Pediatr. (1991) 119:630–8. 10.1016/S0022-3476(05)82418-7 1919897

[32] Ji J Ling XB Zhao Y Hu Z Zheng X Xu Z. A data-driven algorithm integrating clinical and laboratory features for the diagnosis and prognosis of necrotizing enterocolitis. PLoS One. (2014) 9:e89860. 10.1371/journal.pone.0089860 24587080 PMC3938509

