# Supplementary material for: Artificial intelligence in the diagnosis of necrotising enterocolitis from abdominal radiographs: a systematic review and meta-analysis
Source: Front Pediatr. 2026 Jun 18;14:1852254. doi: 10.3389/fped.2026.1852254 (PMC13323305; doi:10.3389/fped.2026.1852254)
Supplement: Supplementary file 1 [file Datasheet1.docx]

**Study Title:** AI in the Diagnosis of Necrotising Enterocolitis from Abdominal Radiographs: A Systematic Review and Meta-analysis

Table S1: Detailed search strategy

| Sr. No | Concept | Search Strategy |
| --- | --- | --- |
| 1 | Newborns / Neonates | "Infant, Newborn"[MeSH Terms] OR "newborn*"[All Fields] OR "neonate*"[All Fields] OR "neonatal"[All Fields] OR "infant*"[All Fields] OR "preterm infant*"[All Fields] OR "premature infant*"[All Fields] |
| 2 | Necrotizing Enterocolitis (NEC) | "Enterocolitis, Necrotizing"[MeSH Terms] OR "necrotizing enterocolitis"[All Fields] OR "necrotising enterocolitis"[All Fields] OR "NEC"[All Fields] |
| 3 | Radiographs / Imaging | "Radiography, Abdominal"[MeSH Terms] OR "Radiography"[MeSH Terms] OR "X-Rays"[MeSH Terms] OR "Diagnostic Imaging"[MeSH Terms] OR "abdominal radiograph*"[All Fields] OR "abdominal x-ray*"[All Fields] OR "plain film*"[All Fields] OR "radiological findings"[All Fields] OR "radiograph*"[All Fields] OR "x-ray*"[All Fields] OR "radiomics"[All Fields] OR "image-based diagnosis"[All Fields] OR "medical imaging"[All Fields] OR "abdominal imaging"[All Fields] |
| 4 | Artificial Intelligence / Machine Learning | "Artificial Intelligence"[MeSH Terms] OR "Machine Learning"[MeSH Terms] OR "Neural Networks, Computer"[MeSH Terms] OR "Deep Learning"[MeSH Terms] OR "Computer-Assisted Diagnosis"[MeSH Terms] OR "artificial intelligence"[All Fields] OR "machine learning"[All Fields] OR "deep learning"[All Fields] OR "neural network*"[All Fields] OR "convolutional neural network*"[All Fields] OR "CNN"[All Fields] OR "AI"[All Fields] OR "ML"[All Fields] OR "radiomics"[All Fields] OR "computer-aided diagnosis"[All Fields] |
| 5 | Combined Search | #1 AND #2 AND #3 AND #4 |


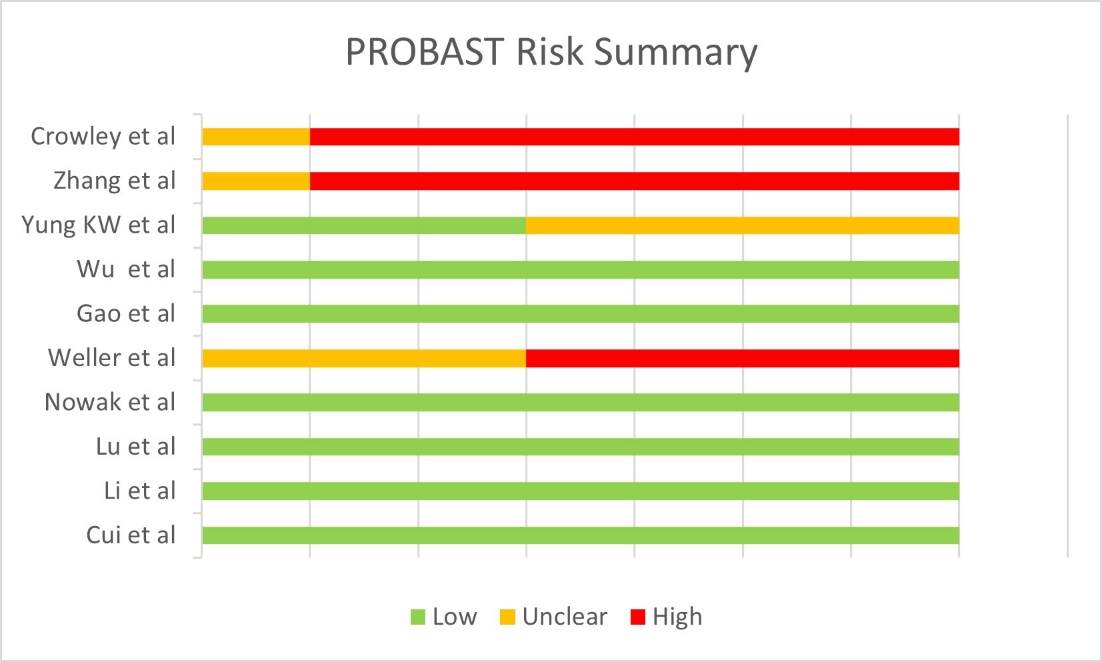


Figure S1: Summary of Risk of Bias assessment of included studies using the modified PROBAST tool


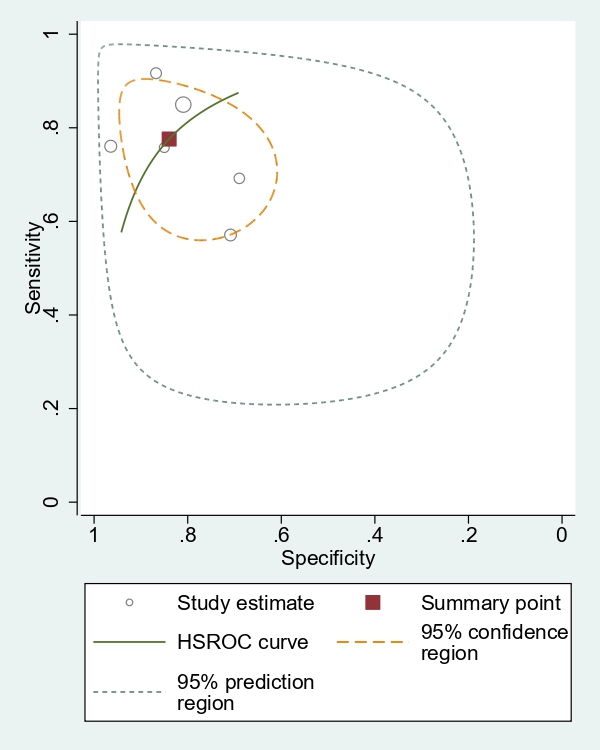


Figure S2: Hierarchical Summary of Receiver Operating Characteristic (HSROC) curve depicting the overall diagnostic accuracy of AI-based models for NEC

Figure S3: Scatter Matrix Plot

Figure S4: Funnel Plot for Publication Bias
